# Antibody-drug conjugates in recurrent or metastatic HNSCC: relevant targets, clinical development, and future prospects

**DOI:** 10.3389/fimmu.2026.1828131

**Published:** 2026-05-21

**Authors:** Hu Zhao, Senxuan Zhang, Fang Wang, Peirong Yu, Jin Li, Lina Jia, Pengfei He, Run Liu, Jian Zhang, KeXin Wang, Rui Han, Hui Huangfu

**Affiliations:** 1 Otorhinolaryngology and Head and Neck Surgery, The First Hospital of Shanxi Medical University, Taiyuan, China; 2 First Clinical Medical College, Shanxi Medical University, Taiyuan, China

**Keywords:** antibody–drug conjugates, cancer, clinical application, R/M HNSCC, targeted therapy

## Abstract

Recurrent or metastatic head and neck squamous cell carcinoma (R/M HNSCC) is a highly aggressive disease entity with poor prognosis. Current systemic treatment regimens, including chemotherapy, targeted therapy, and immune checkpoint inhibitors, provide only limited and often short-lived clinical benefits. As an innovative therapeutic approach, antibody-drug conjugates (ADCs) combine the tumor-targeting specificity of monoclonal antibodies with the potent cytotoxicity of the conjugated payload, enabling selective delivery of highly active drugs to cancer cells. Given the urgent need to improve clinical outcomes for R/M HNSCC, ADCs have emerged as a highly promising therapeutic strategy and are increasingly becoming the focus of translational research and clinical trials, with encouraging signals observed in both early and late-stage trials. This review comprehensively outlines the biological principles, antigen targets, and current clinical development progress of ADCs in treating R/M HNSCC. It begins with a brief explanation of ADC structural principles, followed by a systematic summary of all preclinical and clinical evidence supporting ADC therapy.

## 1 Introduction

Head and neck cancer (HNC) refers to malignant tumors originating in the pharynx, larynx, oral cavity, nasal cavity, and other areas. Among these, head and neck squamous cell carcinoma (HNSCC) accounts for more than 90% of all cases and is the sixth most common cancer worldwide (1). Over the past few decades, its survival rate has gradually increased (2); according to the latest published epidemiological data, the five-year relative survival rate for patients diagnosed with head and neck cancer between 2016 and 2022 has reached approximately 69.9% (3). However, the clinical management of HNSCC is often complicated by the fact that the disease is already at an advanced stage at the time of initial diagnosis. Furthermore, the complex anatomy of the head and neck presents significant challenges in achieving complete surgical resection with adequate margins while preserving critical physiological functions and quality of life. Consequently, despite advances in surgical, radiation, and chemotherapy techniques, a substantial proportion of patients inevitably experience disease progression, leading to recurrent or metastatic (R/M) HNSCC, a late-stage condition characterized by an extremely poor prognosis. The pathogenesis of HNSCC is highly complex and is broadly classified into two distinct subtypes: human papillomavirus (HPV)-negative and HPV-positive. HPV-negative HNSCC is primarily driven by long-term exposure to tobacco and alcohol, typically carries the *TP53* gene mutated, and has a poor clinical prognosis (4). In contrast, HPV-positive HNSCC is primarily driven by the viral oncoproteins E6 and E7, exhibits distinct biological behavior, and generally has a better prognosis (5). At the molecular level, the progression and invasion of HNSCC are driven by key genomic and proteomic alterations. Frequent overexpression of the epidermal growth factor receptor (EGFR) plays a major catalytic role, leading to the excessive activation of key downstream signaling cascades (including the PI3K/AKT/mTOR and RAS/RAF/MEK/ERK pathways) (6). Furthermore, dysregulation of cell cycle control, escape from apoptosis, and an inherently immunosuppressive tumor microenvironment collectively contribute to the high invasiveness of these tumors. Crucially, HNSCC is also characterized by profound intratumoral antigen heterogeneity. This uneven expression of surface targets frequently drives resistance to conventional monospecific therapies and creates severe therapeutic bottlenecks in the R/M stage (7). Consequently, eradicating these heterogeneous tumor populations underscores the urgent need for next-generation ADCs, particularly those leveraging bystander killing effects.

Systemic therapy remains the primary treatment modality for patients with R/M HNSCC. Although the introduction of cetuximab and immune checkpoint inhibitors has reshaped treatment standards, the overall prognosis for this patient population remains dismal, with a median overall survival often falling below 15 months. Furthermore, primary or acquired resistance severely limits clinical benefit, as objective response rates (ORR) to current standard-of-care systemic therapies rarely exceed 20% (8). This severe therapeutic plateau is often driven by immunosuppressive tumor microenvironments and antigen escape (9–11), highlighting the urgent need for novel precision therapeutic strategies capable of overcoming these resistance mechanisms.

Given the persistent limitations of current systemic therapies in R/M HNSCC, the field is pivoting toward next-generation targeting modalities characterized by enhanced specificity and potency. Among these, ADCs have emerged as a promising therapeutic class (12, 13). Structurally, ADCs comprise a monoclonal antibody that selectively targets a tumor-associated antigen linked to a highly potent cytotoxic payload. This design enables the direct delivery of toxic agents to cancer cells while sparing normal tissues (14–16). Following the U.S. Food and Drug Administration (FDA) approval of ado-trastuzumab emtansine (T-DM1) for breast cancer in 2013 (17), six ADCs have been approved for 13 solid tumor indications within the past three years (18–20). This review outlines the structural design principles of ADCs, the primary surface antigens currently under investigation in R/M HNSCC, and the status of their clinical translation.

## 2 Structural components and mechanisms of action of ADCs

ADCs are composed of three core components: a monoclonal antibody, a cytotoxic payload, and a linker. ADCs integrate the target selectivity of monoclonal antibodies with the potent cell-killing capacity of cytotoxic agents, thereby enabling precise tumor targeting combined with high antitumor efficacy (21–23).

Target selection is a critical determinant of the therapeutic efficacy of ADCs (24). An ideal target antigen should exhibit strong tissue specificity, high antigen stability, and efficient internalization capability. Moreover, it should be uniformly and stably expressed on the surface of target tumor cells while showing low or absent expression in normal tissues, ensuring selective antitumor activity while minimizing toxicity to healthy tissues (25–27).

An ideal monoclonal antibody should possess minimal immunogenicity, high specificity, and strong binding affinity for the target antigen to ensure efficient internalization and a prolonged circulating half-life (28). Among the various types of antibodies available, humanized immunoglobulin G (IgG) is the most commonly used ADC antibody due to its lower immunogenicity. IgG has four subtypes: IgG1, IgG2, IgG3, and IgG4. Of the ADC drugs currently on the market, only two use IgG4 as the antibody; the rest all use IgG1. This is because IgG1 is more stable in the systemic circulation, has a longer half-life, and exhibits higher affinity for Fcγ receptors, making it more likely to bind to innate immune cells such as NK cells and macrophages (29). Furthermore, the core structure of IgG provides accessible interchain disulfide bonds and surface lysine residues, which serve as the primary and most reliable sites for linker-payload conjugation (30). In addition, conditionally activated antibodies, also referred to as probody antibodies, represent an emerging strategy. ADCs based on this design are known as probody–drug conjugates (PDCs). Compared with conventional ADCs, the antibody backbone of PDCs is typically immunoglobulin G. Through mechanisms such as the fusion of cleavable linkers and self-masking peptides at the N-terminus, these antibodies exhibit reduced target affinity in normal tissues and are selectively activated within the tumor microenvironment. Upon activation, target binding affinity and payload release are restored, thereby reducing off-target toxicity (31, 32).

The selection of the cytotoxic payload is equally critical and requires agents with potent antitumor activity capable of inducing cell death at picomolar concentrations upon release from the ADCs. Currently, cytotoxic payloads used in ADCs mainly include DNA-damaging agents, microtubule inhibitors, RNA polymerase inhibitors, and topoisomerase inhibitors. To further optimize ADC performance, future research is increasingly focusing on non-cytotoxic payload mechanisms, which significantly broaden the therapeutic horizons beyond traditional chemotherapy. For instance, immune-stimulating antibody conjugates utilize payloads such as Toll-like receptor or STING agonists to stimulate localized innate immune responses, effectively converting immunosuppressive “cold” tumors into highly inflamed “hot” tumors. Another innovative approach involves degrader-antibody conjugates, which leverage targeted protein degraders (e.g., PROTACs) to selectively ubiquitinate and eliminate specific disease-causing proteins, including those traditionally considered “undruggable”. Furthermore, ADCs armed with apoptosis modulators, such as Bcl-xL inhibitors, are being developed to directly and selectively trigger programmed cell death pathways in cancer cells while sparing normal healthy tissues (33–36). In addition, dual-payload ADCs represent another innovative strategy, whereby two synergistic payloads are combined within a single ADC to enhance antitumor efficacy and reduce the risk of drug resistance (37–39).

**Figure 1:**
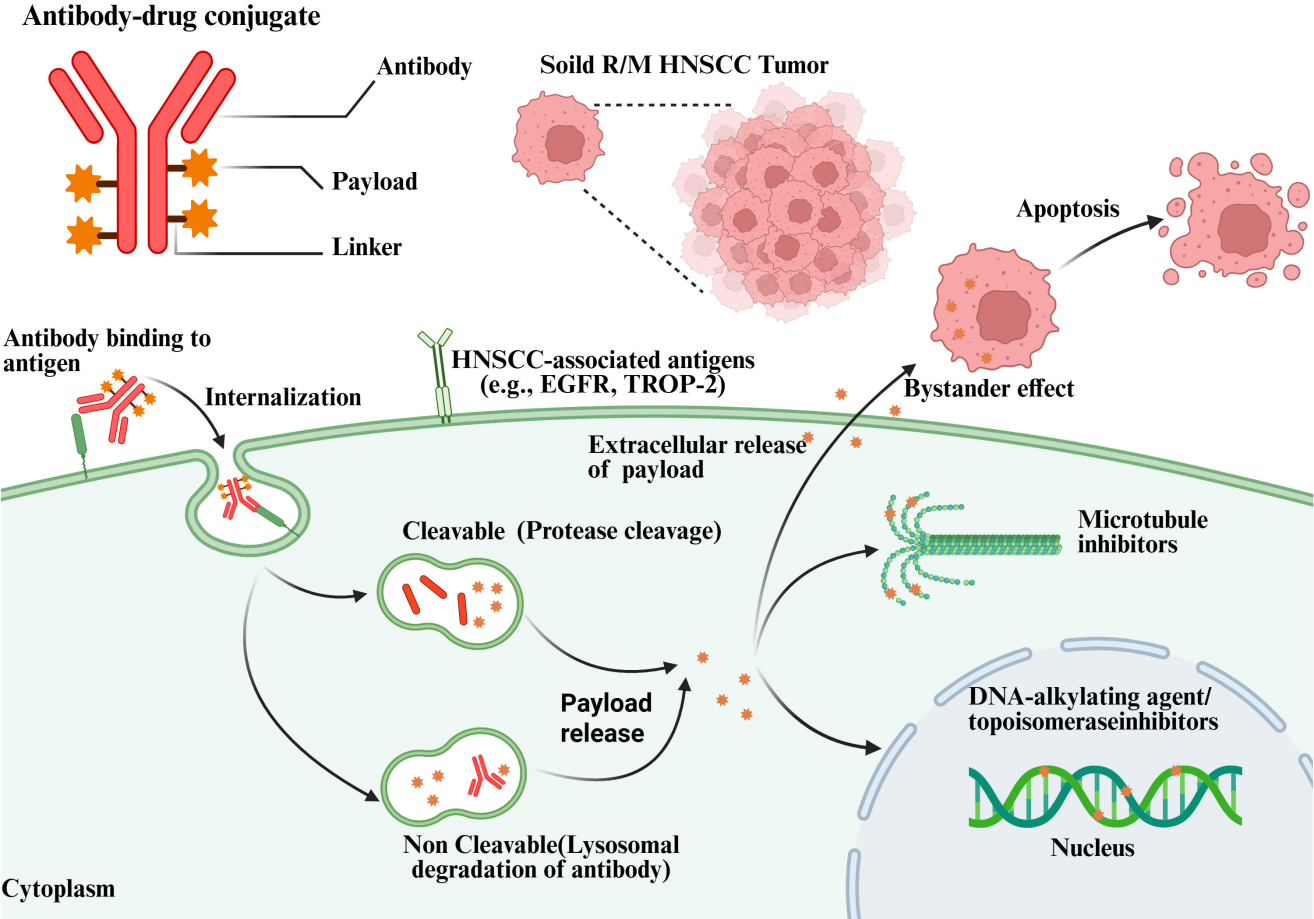
ADCs selectively target tumor-associated antigens expressed on R/M HNSCC cells through antigen-specific monoclonal antibodies. Following antigen binding, ADCs are internalized via receptor-mediated endocytosis and trafficked to lysosomes. Payload release occurs through either enzymatic cleavage of cleavable linkers or lysosomal degradation of non-cleavable linkers. The liberated cytotoxic payloads exert antitumor effects by disrupting microtubule dynamics or inducing DNA damage via alkylation or topoisomerase inhibition, ultimately leading to cell-cycle arrest and apoptosis. In addition, membrane-permeable payloads may diffuse into neighboring tumor cells, producing a bystander killing effect that enhances therapeutic efficacy in heterogeneous tumors. Extracellular payload release may also contribute to antitumor activity within the tumor microenvironment (Created using BioRender.com. MB29LKWIJ0).

Linker selection is a key element in ADC construction, as it directly affects the circulatory stability, pharmacokinetics, and pharmacodynamics of ADC molecules (40). ADC linkers are broadly classified into cleavable and non-cleavable types. Cleavable linkers are rapidly degraded in acidic conditions or within protease-rich lysosomal environments, leading to payload release. If the released payload exhibits sufficient membrane permeability, a bystander killing effect may occur, contributing to enhanced tumor eradication. Bystander cells refer to cells surrounding the target cells, which are typically tumor cells that do not express the target antigen (41). In certain ADCs, payloads are released from target cells or into the extracellular space after antigen binding, allowing them to diffuse into and kill neighboring bystander cells (42, 43) (**Figure 1**). However, not all ADCs exhibit a bystander effect. Current evidence suggests that the presence of a bystander effect may be influenced by several factors, including (1) the extent of ADC internalization following antigen binding (2), linker properties, and (3) the hydrophobicity of the cytotoxic payload (44). In contrast, non-cleavable linkers are biologically inert in circulation and provide greater systemic stability. After internalization into tumor cells, these linkers release cytotoxic molecules only upon lysosomal degradation. However, during this process, amino acid residues remain attached to the linker–payload complex, generating charged metabolites that poorly traverse biological membranes, thereby largely eliminating bystander killing effects. Future research on linker design will focus on achieving an optimal balance between stability and efficacy, with particular emphasis on the development of more stable yet efficiently cleavable linkers (45–47). For example, the tetrapeptide linker used in trastuzumab deruxtecan demonstrates high stability in systemic circulation, significantly reducing off-target toxicity. Upon cellular internalization, this linker is selectively cleaved by lysosomal proteases, and a self-immolative spacer facilitates payload deamination to form a hydroxyl group, enhancing membrane permeability and the bystander effect, ultimately enabling efficient tumor cell killing (45, 48). After entering the bloodstream and reaching the tumor site, ADCs bind to target antigens on the tumor cell surface via the antigen-binding fragment of the monoclonal antibody, forming a complex that is subsequently internalized by tumor cells. Within the tumor microenvironment, linkers are cleaved in lysosomes through proteolytic degradation or acidic conditions, releasing cytotoxic payloads that subsequently disrupt microtubules or damage DNA, thereby exerting potent cytotoxic effects on tumor cells (49).

## 3 Antigenic targets and ADC pharmacologic agents in R/M HNSCC

**Figure 2:**
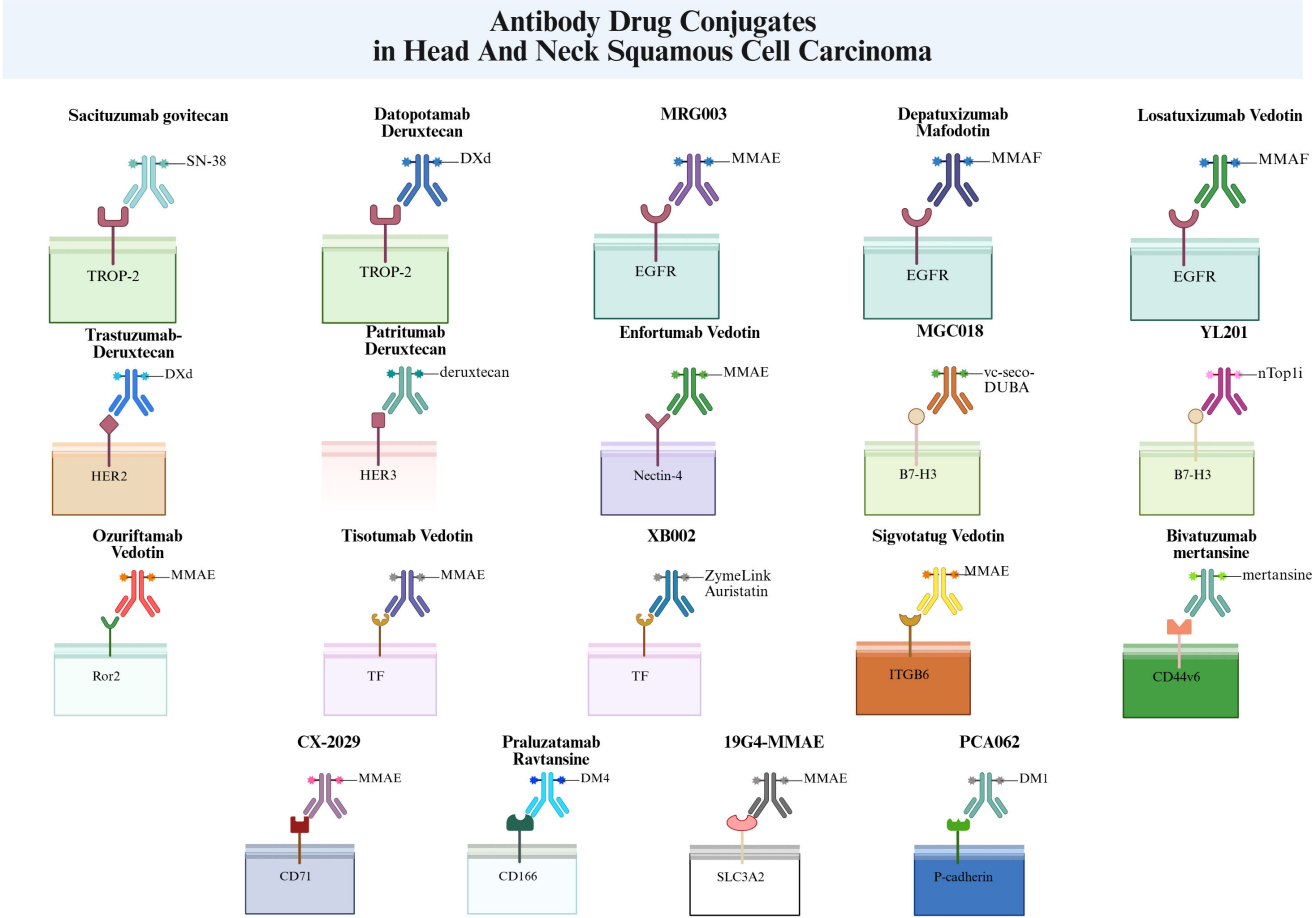
Overview of key cell surface antigens targeted by ADCs in recurrent/metastatic head and neck squamous cell carcinoma. This schematic highlights major targets and corresponding ADCs (created by BioRender.com. WL29LKWITL).

During the development of ADCs, multiple antigen targets exist. This section briefly reviews these antigen targets and all ADC drugs currently under investigation in the R/M HNSCC field (**Figure 2**).

### 3.1 ADCs targeting trophoblast cell-surface antigen 2

TROP-2 is a transmembrane glycoprotein encoded by the *TACSTD2* gene (50). Transcriptomic data, including those from The Cancer Genome Atlas(TCGA; https://portal.gdc.cancer.gov/), have confirmed that TROP-2 is significantly overexpressed in R/M HNSCC relative to adjacent normal tissues (51). This overexpression is closely associated with the activation of oncogenic signaling pathways, such as PI3K/AKT and MAPK/ERK, which subsequently drive tumor cell proliferation, invasion, and epithelial-mesenchymal transition. While the complexity of these downstream signaling networks limits the efficacy of small-molecule inhibitors—since blocking a single target or pathway may not sufficiently suppress TROP-2 function, ADCs can effectively exploit this high expression. By utilizing TROP-2 as a portal for antibody-mediated internalization, ADCs deliver cytotoxic payloads that disrupt critical cellular machinery, such as nucleic acids or microtubules, a therapeutic strategy that has been substantiated by emerging evidence (52–55).

#### 3.1.1 Sacituzumab govitecan

SG comprises a humanized anti-TROP-2 monoclonal antibody conjugated to the topoisomerase I inhibitor SN-38 via a hydrolyzable CL2A linker, a design featuring a high drug-to-antibody ratio and a potent bystander killing effect (56). Currently approved for breast and urothelial carcinomas (57–59), SG was evaluated in the single-arm, basket Phase II TROPiCS-03 study (NCT03964727) based on TROP-2 overexpression in HNSCC and the unmet need following platinum and PD-1 blockade failure. Among a relatively small cohort of 43 patients with recurrent or refractory disease, the objective response rate (ORR) was 16% (which, alongside a median progression-free survival (PFS) of 4.1 months, compares favorably against the historical 10–15% ORR and 2–3 months PFS typically seen with standard salvage chemotherapy), with a clinical benefit rate of 28%. The median duration of response (DOR), PFS, and overall survival (OS) were 4.2, 4.1, and 9.0 months, respectively. The most common treatment-emergent adverse events (TEAEs) included diarrhea, nausea, and neutropenia (47% each). Grade ≥3 TEAEs occurred in 58% of patients; three deaths were reported, one of which (infectious shock) was considered treatment-related(**Table 1**) (60). While these preliminary efficacy signals are encouraging in a heavily pretreated population, larger randomized controlled trials are required to definitively establish its superiority over current standard-of-care regimens.

**Table 1:** Clinical development of ADCs in R/M HNSCC.

Drug	Target	NCT	Phase	N	ORR	mPFS	mOS	Line of therapy	TEAE
Sacituzumab govitecan (60)	TROP2	NCT03964727	II	43	16%	4.1	9.0	Post-PD-1/L1	Diarrhea (47%), nausea (47%), and neutropenia (47%)
MRG003 (67)	EGFR	NCT0486834	II	61	40%	2.8	11.8	Advanced R/M (Salvage)	Hyponatremia, leukopenia, neutropenia, elevated aspartate aminotransferase, febrile neutropenia.
Losatuxizumab Vedotin (71)	EGFR	NCT02365662	I	5	-	-	-	Heavily Pretreated	Fatigue (44.4%), diarrhea (24.4%), nausea (22.2%), rash (22.2%), and pruritus (20.0%)
patritumab deruxtecan (87)	HER3	NCT06172478	II	40	-	-	-	Advanced R/M (Salvage)	Research is currently underway.
Enfortumab Vedotin (94)	Nectin-4	NCT04225117	II	46	23.90%	3.9	6.0	Heavily Pretreated	Hair loss, fatigue, and peripheral neuropathy
YL20 (99)	B7H3	NCT05434234	I	20	40.80%	5.9	-	Advanced R/M (Salvage)	Leukopenia, anemia, and neutropenia; anorexia, nausea, and hypoalbuminemia.
Ozuriftamab Vedotin (102)	ROR2	NCT05271604	II	31	-	-	-	Post-PD-1/L1	Fatigue, nausea, anemia, decreased appetite, and diarrhea. A total of 19 patients experienced Grade 3–4 TEAEs; the most common (each 10%) were anemia, hyponatremia, and hypoxemia.
Tisotumab Vedotin (108)	TF	NCT03485209	II	31	16%	4.2	9.4	Post-PD-1/L1	The most common adverse events (≥10%) were anemia, pneumonia, and dyspnea.
Sigvotatug Vedotin (113)	ITGB6	NCT04389632	I	20	20%	-	-	Post-PD-1/L1	Fatigue, and the most common Grade 3 or higher TEAE was neutropenia.
Bivatuzumab mertansine (117)	CD44v6	Not found	I	31	10%	-	-	Advanced R/M (Salvage)	Maculopapular rash, focal vesicle formation, and skin desquamation
CX2029 (121)	CD71	PROCLAIM-CX-2029	I	8	12%	-	-	Post-PD-1/L1	IRR (87%), anemia (67%), nausea (36%), decreased appetite (27%), and fatigue (27%).
Praluzatamab Ravtansine (124)	CD166	NCT03149549	II	9	11%	-	-	≥2L (Post-IO)	Keratitis (9%), elevated aspartate aminotransferase (8%), elevated alanine aminotransferase (5%), and anemia (5%).
PCA062 (130)	P-cadherin	NCT02375958	I	6	20%	-	-	Post-PD-1/L1	Elevated aspartate aminotransferase, thrombocytopenia, and anemia
Izalontamab (133)	EGFR×HER3	NCT04603287	Ib	9	4%	1.9	3.6	Advanced R/M (Salvage)	Rash (42%), paronychia (25%), and infusion-related reactions (23%).
Izalontamab brengitecan (131)	EGFR×HER3	NCT06118333	III	386	54.6%	8.38	12.3	Advanced R/M (Salvage)	Neutropenia, anemia, gastrointestinal reactions (nausea, vomiting), fatigue, and manageable bone marrow suppression associated with topoisomerase I inhibitors.

*ORR* overall response rate, *mPFS* median progression-free survival, *mOS* median overall survival, *TEAE* treatment-emergent adverse event

#### 3.1.2 Datopotamab Deruxtecan (Dato-DXd, DS-1062a)

Dato-DXd is composed of a recombinant humanized anti–TROP-2 IgG1 monoclonal antibody conjugated to a topoisomerase I inhibitor payload (DXd) via a tetrapeptide-based cleavable linker attached to cysteine residues between disulfide bonds of the antibody (61). The pharmacologic activity and mechanism of action of Dato-DXd have been investigated in human pharyngeal carcinoma cell lines (FaDu) and xenograft mouse models (utilizing cell lines such as NCI-N87 and Calu-3), including patient-derived xenograft (PDX) models. Safety evaluations conducted in rats and cynomolgus monkeys revealed an acceptable and well-tolerated safety profile; notably, Dato-DXd induced only slight intestinal or hematopoietic toxicities without severe bone marrow suppression even at high doses, highlighting its manageable toxicity. Dato-DXd demonstrated significant inhibition of tumor cell growth in TROP-2–high–expressing cell lines (62). While these preclinical findings establish a robust mechanistic rationale, the efficacy of Dato-DXd in R/M HNSCC has not yet been fully characterized in clinical cohorts. Consequently, its therapeutic potential and safety profile relative to current standard-of-care regimens must be rigorously validated in future clinical trials.

### 3.2 ADCs targeting epidermal growth factor receptor

EGFR, a transmembrane glycoprotein belonging to the receptor tyrosine kinase family, is encoded by a gene located on chromosome 7 (63). Upon ligand binding, EGFR undergoes a conformational change that promotes receptor homo- or heterodimerization and the subsequent autophosphorylation of intracellular tyrosine residues, thereby recruiting effector proteins containing SH2 or PTB domains. This process activates critical downstream signaling cascades—including the PI3K/AKT/mTOR, RAS/RAF/MEK/ERK, and JAK/STAT pathways (64)—which directly drive the proliferation, survival, and metastasis of HNSCC cells (65). Given its pivotal role in the pathogenesis of HNSCC, EGFR has emerged as an ideal target for the development of ADCs, including MRG003, depatuxizumab mafodotin, and losatuxizumab vedotin.

#### 3.2.1 MRG003

MRG003 is composed of a humanized anti-EGFR IgG1 monoclonal antibody conjugated to monomethyl auristatin E (MMAE) via a valine-citrulline linker (66). In a Phase I study (NCT04868344) evaluating the 2.5 mg/kg dose in patients with previously treated, EGFR-positive advanced HNSCC, the agent elicited an ORR of 40% and a disease control rate (DCR) of 100%. When benchmarked against the dismal 10%–15% ORR historically achieved with standard salvage regimens in similar refractory populations, this 40% response rate represents a highly encouraging clinical signal. The median DOR, PFS, and OS were 5.6, 2.8, and 11.8 months, respectively. Regarding safety, 89% of adverse events were considered treatment-related, predominantly Grade 1–2. Grade ≥3 treatment-related adverse events (TRAEs) occurred in 31% of patients, primarily involving hyponatremia, leukopenia, neutropenia, and elevated aspartate aminotransferase (**Table 1**) (67). However, given the single-arm design of this early-phase trial, these robust initial findings await definitive validation through larger, randomized controlled trials.

#### 3.2.2 Depatuxizumab Mafodotin (ABT-414)

ABT-414 consists of the antibody ABT-806 conjugated to monomethyl auristatin F (MMAF) via a non-cleavable maleimidocaproyl linker attached to interchain cysteines, featuring an average drug-to-antibody ratio of 3.8 (68). While clinical data are currently lacking, preclinical studies evaluated its efficacy in EGFR-overexpressing HNSCC cell lines (UMSCC47 and FaDu). In xenograft models, ABT-414 treatment resulted in significant tumor growth inhibition in the FaDu cohort (P = 0.023). Conversely, although a reduction in growth rate was observed in the UMSCC47 model, the difference did not reach statistical significance (P = 0.176). These findings highlight the potential efficacy of ABT-414 in specific EGFR-high tumors (69). However, because the current level of evidence remains strictly preclinical, it is not yet possible to benchmark its safety or therapeutic efficacy against established standard-of-care clinical regimens in R/M HNSCC.

#### 3.2.3 Losatuxizumab Vedotin(ABBV-221)

ABBV-221 is a next-generation EGFR-targeting ADC comprising an affinity-matured ABT-806 antibody conjugated to MMAF. Compared to its predecessor, depatuxizumab mafodotin, ABBV-221 retains tumor selectivity while demonstrating superior binding affinity, enhanced *in vitro* potency, and improved antitumor activity in wild-type EGFR xenografts, with a significantly reduced incidence of corneal toxicity (70). In a Phase I study (NCT02365662) including five HNSCC patients, a confirmed partial response (PR) was observed in one patient exhibiting elevated levels of EGFR and its ligands (amphiregulin/epiregulin), supporting the therapeutic validity of targeting EGFR in this setting (**Table 1**) (71). However, given this exceptionally small and highly preliminary sample size, this anecdotal response serves primarily as biological proof-of-concept.

### 3.3 ADCs targeting human epidermal growth factor receptor 2

HER2 is a member of the ErbB receptor tyrosine kinase family (72). Unlike other family members, HER2 lacks a ligand-binding domain but serves as the preferred dimerization partner (73). It acts as a potent signal amplifier to activate downstream pathways, such as PI3K/AKT and RAF/MEK/MAPK, thereby driving tumor cell survival, proliferation, and cell cycle progression (74). In HNSCC, reported rates of HER2 overexpression range from 0% to 47%, and elevated expression is significantly associated with poor prognosis, increased recurrence rates, and reduced OS (75). Given its established role as an oncogenic driver, HER2 has emerged as a key therapeutic target for ADCs in HNSCC, including trastuzumab deruxtecan (T-DXd).

#### 3.3.1 T-DXd

T-DXd comprises a humanized anti-HER2 IgG1 antibody conjugated to the topoisomerase I inhibitor DXd via a cleavable tetrapeptide linker (76). Mechanistically, T-DXd disrupts HER2 signaling, mediates ADCC, and undergoes lysosomal cleavage to release a membrane-permeable payload that induces DNA damage and apoptosis (77). While FDA-approved for various solid tumors (78), clinical data in HNSCC remain limited. However, preclinical studies utilizing HNSCC cell lines (UMSCC-47, FaDu, UMSCC-1) demonstrated that T-DXd exerts potent cytotoxicity even in low-HER2 expressing models. Notably, in FaDu cells, T-DXd significantly inhibited growth where trastuzumab and T-DM1 failed. These findings were further validated *in vivo* using cell line-derived xenograft (CDX) murine models utilizing the FaDu and UMSCC-47 cell lines. In these HNSCC CDX models, T-DXd elicited superior antitumor activity and significant tumor regression compared to vehicle controls (P = 0.0012 for FaDu). Furthermore, safety evaluations in these preclinical models demonstrated that T-DXd induced profound tumor inhibition without causing any abnormalities in the general condition or body weight of the mice, confirming a highly tolerable safety profile that strongly supports its continued clinical translation for low-HER2 R/M HNSCC (79).

### 3.4 ADCs targeting human epidermal growth factor receptor 3

HER3 is encoded by a gene located on chromosome 12. Distinguished from other ErbB family members by its lack of intrinsic kinase activity and inability to form homodimers, HER3 functions primarily by forming heterodimers with other family members. It utilizes its intracellular C-terminal tail to recruit and activate the PI3K/AKT pathway, thereby playing a pivotal role in tumorigenesis (80). In HNSCC, HER3 not only drives tumor progression (81) but also serves as a crucial mediator of acquired resistance to EGFR inhibitors (82). Owing to its kinase-impaired nature, which limits the efficacy of traditional small-molecule inhibitors, HER3 has emerged as an ideal target for ADCs to overcome drug resistance and enable multimodal precision therapy.

#### 3.4.1 Patritumab deruxtecan HER3-DXd

HER3-DXd comprises an anti-HER3 IgG1 monoclonal antibody (patritumab) conjugated to a topoisomerase I inhibitor payload (deruxtecan) via a stable, cleavable tetrapeptide linker (83). Mechanistically, HER3-DXd undergoes receptor-mediated internalization and lysosomal cleavage, releasing the payload to induce DNA damage while exerting a bystander effect (84). While significant efficacy has been demonstrated in advanced breast cancer and EGFR-mutated NSCLC (85, 86), its application in HNSCC remains under investigation. Currently, an ongoing Phase II trial (NCT06172478) is enrolling 40 patients with unresectable or metastatic disease to evaluate the efficacy and safety of HER3-DXd administered at 5.6 mg/kg every three weeks (**Table 1**) (87). Although efficacy data are pending, the future readouts from this preliminary cohort will be pivotal in establishing the initial level of clinical evidence.

### 3.5 ADCs targeting Nectin-4

Nectin-4, a cell surface glycoprotein belonging to the immunoglobulin superfamily, is aberrantly upregulated in various malignancies and is closely associated with tumor proliferation, metastasis, and drug resistance (88). Mechanistically, Nectin-4 has been shown to facilitate the escape of cancer cells from anoikis by activating the integrin β4/SHP-2/c-Src signaling axis (89). In HNSCC, immunohistochemical analysis revealed a Nectin-4 positivity rate as high as 86.2%, with 32.7% of cases exhibiting moderate-to-high expression (90). This widespread, tumor-specific expression profile establishes Nectin-4 as an ideal therapeutic target in HNSCC, serving as the rationale for the development of ADCs such as enfortumab vedotin.

#### 3.5.1 Enfortumab vedotin

EV comprises a fully human anti-Nectin-4 IgG1 monoclonal antibody conjugated to the microtubule-disrupting agent MMAE via a protease-cleavable linker (DAR 4.0) (91), and is currently approved for urothelial carcinoma (92). Mechanistically, EV undergoes internalization and releases MMAE to disrupt microtubule dynamics, inducing apoptosis (93). A Phase II study (NCT04225117) evaluated EV (1.25 mg/kg administered on days 1, 8, and 15) in 46 patients with unresectable R/M HNSCC. The ORR and DCR were 23.9% (favorably exceeding the historical ~10–15% ORR of standard salvage therapies) and 56.5%, respectively. The median DOR, PFS, and OS were 9.4, 3.9, and 6.0 months. Common TRAEs included alopecia (28.3%), fatigue (26.1%), and peripheral sensory neuropathy (23.9%). These data confirm the clinically meaningful activity of EV in patients with prior progression on PD-1/L1 inhibitors (**Table 1**) (94), though randomized trials are needed to elevate this preliminary level of evidence.

### 3.6 ADCs targeting B7-H3

B7-H3, also known as CD276, is a type I transmembrane immunomodulatory protein. While B7-H3 mRNA is detectable at low levels in most normal tissues, its expression is significantly upregulated in HNSCC, rendering it a highly specific therapeutic target (95). Clinically, B7-H3 overexpression correlates with aggressive clinicopathological features in HNSCC, including increased tumor burden, advanced clinical stage, and poor survival outcomes. Furthermore, B7-H3 contributes to an immunosuppressive tumor microenvironment, as its expression is positively associated with the infiltration of M2 macrophages and myeloid-derived suppressor cells (96). Currently, investigational ADCs targeting B7-H3, such as YL201 and MGC018, are under evaluation in this setting.

#### 3.6.1 MGC018

MGC018 comprises a humanized anti-B7-H3 IgG1 monoclonal antibody conjugated to the DNA-alkylating payload vc-seco-DUBA via a cleavable linker, with a drug-to-antibody ratio of approximately 2.7 (97). Exhibiting both direct cytotoxicity and a bystander effect, MGC018 demonstrated profound efficacy in HNSCC patient-derived xenograft (PDX) models. Treatment at 3 mg/kg resulted in a 98% reduction in tumor volume compared to vehicle, with a favorable safety profile characterized by the absence of significant toxicity. These preclinical findings support the continued development of MGC018 as a therapeutic agent for HNSCC (98).

#### 3.6.2 YL201

Developed using the TMALIN tumor microenvironment-activatable platform, YL201 comprises a human anti-B7-H3 monoclonal antibody conjugated to a novel topoisomerase I inhibitor via a protease-cleavable linker (DAR 8.0). A global multicenter Phase I trial (NCT05434234) evaluating YL201 in 312 patients with advanced solid tumors, including 20 with HNSCC, reported an ORR of 40.8% (a robust signal compared to the ~10–15% historical ORR of standard salvage therapy) and a DCR of 83.6%. The mPFS and mDOR were 5.9 and 6.3 months, respectively. TRAEs occurred in 97.1% of patients, most commonly leukopenia, anemia, neutropenia, anorexia, nausea, and hypoalbuminemia. These findings support the continued clinical development of YL201 (**Table 1**) (99), pending validation in larger randomized trials to formally establish its efficacy against the standard of care.

### 3.7 ADCs targeting receptor tyrosine kinase-like orphan receptor 2

ROR2 is a transmembrane protein that plays a critical role in embryonic development (100). While ROR2 expression is minimal in adult healthy tissues, it is significantly upregulated in various malignancies, including HNSCC, where it drives tumor progression by promoting cell migration and invasion (101). This high tumor specificity renders ROR2 a promising therapeutic target for ADC development. Currently, the ROR2-targeting ADC ozuriftamab vedotin (BA3021) is under clinical evaluation and was granted Fast Track designation by the FDA in July 2024 for the treatment of recurrent or metastatic HNSCC.

#### 3.7.1 Ozuriftamab vedotin

Ozuriftamab vedotin (BA3021) is a conditionally active ADC designed to bind ROR2 selectively within the acidic tumor microenvironment. It comprises a pH-responsive IgG1 antibody (BA302) conjugated to MMAE via a protease-cleavable linker (DAR 4:1) (102). A Phase II trial evaluated BA3021 (1.8 mg/kg every two weeks) in R/M HNSCC patients progressing on PD-1 inhibitors. Among 31 patients, 8 achieved a response. Common TEAEs included fatigue, nausea, anemia, decreased appetite, and diarrhea. Grade 3–4 TEAEs occurred in 19 patients, predominantly anemia, hyponatremia, and hypoxia (10% each). These data demonstrate promising antitumor activity with a manageable safety profile (**Table 1**) (103).

### 3.8 ADCs targeting full-length tissue factor

TF is a transmembrane receptor that functions as a cofactor for Factor VII/FVIIa. While an alternatively spliced, soluble isoform exists, the membrane-bound full-length TF serves as the primary target for ADC intervention (104). Driven by mechanisms such as hypoxic microenvironments, oncogene activation, and loss of tumor suppressors, TF is aberrantly overexpressed in malignancies compared to normal tissues. In HNSCC, membranous TF expression is predominant and has been detected in at least 75% of cases (105). This high prevalence of surface expression provides a compelling rationale for ADC development. Currently, TF-targeting ADCs, including tisotumab vedotin, XB002, and InnovaTV 207, are under clinical investigation.

#### 3.8.1 Tisotumab vedotin

TV comprises an anti-tissue factor (TF) monoclonal antibody conjugated to MMAE via a protease-cleavable valine-citrulline linker (106). Mechanistically, internalization and lysosomal cleavage release MMAE, which disrupts microtubule polymerization to induce G2/M arrest, apoptosis, and a bystander effect (107). Currently approved for cervical cancer (108), TV was evaluated in a Phase II HNSCC study (N = 31; 2 mg/kg Q3W). Results demonstrated an ORR of 16% (which sits at the upper bound of the historical 10–15% ORR observed with standard salvage regimens) and a DCR of 58.1%, with mPFS and mOS of 4.2 and 9.4 months, respectively. Common adverse events(≥10%) included anemia, pneumonia, and dyspnea. These data indicate a manageable safety profile and preliminary antitumor activity (**Table 1**) (109), highlighting the need for larger, randomized trials to definitively establish its clinical standing.

#### 3.8.2 XB002

XB002 is an anti-TF ADC engineered to minimize bleeding risks, comprising the anti-TF mAb 25A3 conjugated to the ZymeLink Auristatin payload (zovodotin) via a protease-cleavable linker. Preclinical PDX models demonstrated that a single intravenous dose (10 mg/kg) inhibited tumor growth across multiple indications, achieving complete regression in HNSCC and cervical cancer cohorts within 30 days. Unlike tisotumab vedotin and MRG004A, XB002 did not alter coagulation parameters, indicating a superior safety profile regarding bleeding risks and supporting its clinical translation (110).

### 3.9 ADCs targeting Integrin beta-6

ITGB6 is characterized by strict pairing specificity, forming a transmembrane heterodimer exclusively with the αv subunit. The β6 subunit contains highly conserved cytoplasmic domains that are essential for mediating intracellular signaling interactions (111). In HNSCC, ITGB6 is significantly upregulated compared to normal tissues. Clinically, high ITGB6 expression is strongly correlated with aggressive tumor phenotypes, including invasion and metastasis, as well as reduced median survival. This tumor-associated overexpression profile positions ITGB6 as a compelling emerging target for ADC development (112). Currently, sigvotatug vedotin (SGN-B6A) is the leading investigational ADC directed against this target.

#### 3.9.1 SGN-B6A

SGN-B6A is an ADC targeting ITGB6, in which a protease-cleavable linker is used to deliver the cytotoxic payload MMAE to tumor cells. The antibody component of SGN-B6A specifically recognizes integrin β6 and does not bind other members of the αv integrin family. Preclinical *in vitro* assessments demonstrated its target-dependent cytotoxicity across various integrin β6-expressing carcinoma cell lines. Its *in vivo* efficacy in head and neck cancer was specifically evaluated utilizing CDX models established with the human pharyngeal carcinoma cell line, Detroit 562. In this CDX model, weekly administration of SGN-B6A at a dose of 3 mg/kg for three consecutive weeks resulted in tumor volume reductions exceeding 30% when compared to untreated controls and nonbinding control ADCs. Furthermore, nonclinical safety evaluations in cynomolgus monkeys revealed a highly favorable safety profile; SGN-B6A was well-tolerated with a highest non-severely toxic dose of 5 mg/kg weekly. The primary safety outcomes were manageable and reversible hematologic changes, such as neutropenia, without severe off-target toxicities, strongly supporting its therapeutic potential and clinical translation for R/M HNSCC (113). In a phase I clinical trial evaluating the safety and antitumor activity of SGN-B6A in R/M HNSCC (NCT04389632), the ORR in the R/M HNSCC cohort was 20% (a meaningful improvement over the historical 10–15% ORR typical of standard salvage therapies), with a median DOR of 5.5 months. The most common TEAE was fatigue, while the most frequent grade ≥3 TEAE was neutropenia. Peripheral sensory neuropathy and pneumonia were the most common TEAEs leading to treatment discontinuation. Overall, SGN-B6A demonstrated an acceptable safety profile and encouraging preliminary antitumor activity with durable responses in heavily pretreated patients (**Table 1**) (114), though larger randomized trials are ultimately necessary to confirm its comparative efficacy against standard-of-care regimens.

### 3.10 ADCs targeting CD44v6

CD44 variant 6 (CD44v6) is a major splice variant of the transmembrane glycoprotein CD44, encoded by a gene on chromosome 11p13. Recognized as a critical driver of metastasis, CD44v6 facilitates tumor progression by enabling cancer cells to mimic lymphocytes, thereby evading immune surveillance and promoting lymphatic and hematogenous dissemination in HNSCC (115). Given its pivotal role in tumorigenesis and invasion, CD44v6 serves as a specific target for ADC development. Accordingly, bivatuzumab mertansine, an ADC directed against this variant, has entered Phase I clinical evaluation.

#### 3.10.1 Bivatuzumab mertansine

Bivatuzumab mertansine is a CD44v6-targeting ADC comprising bivatuzumab conjugated to the antitubulin agent mertansine via a disulfide bond. Upon internalization and cleavage, mertansine inhibits tubulin polymerization, inducing mitotic arrest and apoptosis. In a safety study involving seven HNSCC patients (20–140 mg/m² weekly), five achieved stable disease (SD); however, the trial was terminated following one case of fatal toxic epidermal necrolysis (116). Another Phase I study enrolling 31 HNSCC patients (starting at 25 mg/m²) reported three partial responses (PR), yielding an approximate ORR of 10%, which merely mirrors the baseline historical outcomes of standard salvage regimens. Notably, 80% of patients experienced skin-related adverse events, including erythema, maculopapular rash, blistering, and desquamation (**Table 1**) (117). Consequently, this prohibitive safety profile, coupled with modest baseline-level efficacy, effectively precluded its further clinical development and comparative evaluation against standard-of-care options.

### 3.11 ADCs targeting CD71

CD71 (Transferrin receptor 1), encoded by the *TFRC* gene, is a transmembrane receptor essential for cellular iron uptake. Driven by the heightened metabolic iron demands of rapidly proliferating cells, CD71 is markedly upregulated in malignancies compared to normal tissues (118). In HNSCC, elevated CD71 expression levels are positively correlated with increased tumor burden, lymph node metastasis, and advanced disease severity (119). This tumor-specific metabolic dependency validates CD71 as a viable therapeutic target. Currently, CX-2029, a novel therapeutic agent targeting CD71, is under clinical development.

#### 3.11.1 CX2029

Probody therapeutic conjugates (Pb-Txs) are conditionally active ADCs designed to remain inactive until protease-activated within the tumor microenvironment, enabling safe targeting of ubiquitously expressed antigens. CX-2029 is a CD71-targeting Pb-Tx conjugated to MMAE via a maleimido-caproyl-valine-citrulline-p-aminobenzyloxycarbonyl linker (DAR 2.0) (120). A Phase I trial enrolling 45 patients (including 8 with HNSCC) treated with CX-2029 every three weeks reported an ORR of 12%. Common TEAEs included infusion-related reactions (IRR, 87%), anemia (67%), nausea (36%), decreased appetite, and fatigue. This first-in-human study validated CD71 as a viable target and demonstrated that the Pb-Tx platform effectively mitigates on-target/off-tumor toxicity, showing good tolerability and meaningful clinical activity (**Table 1**) (121).

### 3.12 ADCs targeting CD166

Activated leukocyte cell adhesion molecule (CD166/ALCAM) is a transmembrane glycoprotein of the immunoglobulin superfamily that mediates critical cell-cell interactions. Functioning as a regulator of tumor progression, CD166 modulates cell proliferation, adhesion, migration, and invasion, with overexpression frequently correlating with poor clinical prognosis (122). In HNSCC, CD166 expression has been reported in up to 70.3% of cases (123). This high prevalence of surface expression provides a compelling rationale for validating CD166 as a therapeutic target for ADC intervention.

#### 3.12.1 Praluzatamab ravtansine (CX-2009)

CX-2009 is a CD166-targeting, conditionally active Probody ADC comprising a monoclonal antibody conjugated to DM4 via a protease-cleavable linker (DAR ~3.5). A single-arm Phase I/II study (NCT03149549) evaluating the agent (administered Q3W) in a small cohort of 9 HNSCC patients reported an ORR of 11% (which falls strictly within the historical 10–15% baseline ORR of standard salvage regimens). The most common Grade ≥3 TRAEs included keratitis (9%), elevated aspartate aminotransferase (AST) (8%), elevated alanine aminotransferase (ALT) (5%), and anemia (5%). These results validate the viability of CD166 as a first-in-class therapeutic target (**Table 1**) (124), though larger randomized cohorts are required to formally establish its clinical efficacy relative to current standard-of-care options.

### 3.13 ADCs targeting Solute carrier family 3 member 2

SLC3A2, also known as the CD98 heavy chain (CD98hc), is a type II transmembrane protein that complexes with light chains to form heterodimeric amino acid transporters essential for cell survival. In HNSCC, SLC3A2 overexpression drives tumor progression and correlates with poor prognosis by activating the mTOR pathway, enhancing amino acid metabolism and DNA repair, and suppressing oxidative stress and autophagy (125). This pivotal role in regulating tumor metabolism renders SLC3A2 a promising therapeutic target. Currently, 19G4-MMAE, a novel ADC directed against SLC3A2, is under development.

#### 3.13.1 19G4-MMAE

First reported in 2024, 19G4-MMAE is a novel SLC3A2-targeting ADC comprising a humanized IgG1 monoclonal antibody conjugated to MMAE. Preclinical evaluations demonstrated potent anti-proliferative effects in SLC3A2-positive HNSCC cell lines (Including SCC15, NPC/HK1, and FADU), while no significant cytotoxicity was observed in low-SLC3A2-expressing cells like C666-1. The *in vivo* efficacy was further validated using CDX murine models established with the SCC15 cell line. In these models, 19G4-MMAE treatment elicited significant tumor growth inhibition compared to vehicle controls (PBS). Safety assessments in these CDX models revealed a highly tolerable profile; 19G4-MMAE was well-tolerated at therapeutic dosages without causing significant weight loss or pathological damage to vital organs such as the heart and liver, highlighting its potential as a promising therapeutic candidate for HNSCC (126).

### 3.14 ADCs targeting P-Cadherin

P-cadherin is a calcium-dependent cell–cell adhesion molecule that plays a critical role in maintaining cellular structural integrity, morphology, proliferation, and differentiation. Through its calcium-mediated adhesive function, P-cadherin preserves tight intercellular connections. Reduced expression leads to decreased adhesion, facilitating tumor invasion and metastasis. *In vitro* studies utilizing hepatocellular carcinoma cell lines (specifically Huh7, HepG2, and Hep3B) have demonstrated that suppression of P-cadherin expression promotes cancer cell proliferation (127). However, its role in tumor progression remains controversial. The study by Liu et al. reported significant overexpression of P-cadherin in R/M HNSCC samples, and P-cadherin knockdown reduced cell proliferation, migration, and invasion, whereas overexpression enhanced these malignant behaviors (128). PCA062 is an ADC targeting P-cadherin in R/M HNSCC.

#### 3.14.1 PCA062

PCA062 is a P-cadherin-targeting ADC comprising a fully human monoclonal antibody conjugated to DM1 via a non-cleavable SMCC linker (129). In a Phase I trial (NCT02375958) enrolling 6 HNSCC patients, one partial response (PR) was observed among five evaluable patients (ORR 20%). The incidence of Grade ≥3 TRAEs across all cohorts was 28%, predominantly elevated AST, thrombocytopenia, and anemia. Clinical development was subsequently terminated due to limited antitumor activity at the maximum tolerated dose (**Table 1**) (130).

## 4 Bispecific antibody–drug conjugates

BsADCs represent a sophisticated evolution in the ADC landscape, designed to address the limitations of conventional monospecific ADCs, such as target heterogeneity and resistance driven by antigen escape. Unlike standard ADCs, BsADCs utilize a bispecific antibody backbone capable of simultaneously binding to two different antigens or two distinct epitopes on the same antigen. One primary strategy is dual-antigen targeting (e.g., EGFR×HER3), which enhances tumor-binding specificity and enables the simultaneous blockade of compensatory signaling pathways, thereby overcoming primary or acquired resistance (131). Another innovative approach is biparatopic targeting, where the ADC binds to non-overlapping epitopes of a single antigen, a mechanism that can trigger superior receptor clustering, accelerated internalization, and more efficient lysosomal trafficking compared to monospecific antibodies (132). By integrating multi-target synergy with potent cytotoxic delivery, BsADCs offer a promising modality to expand the therapeutic window and enhance clinical efficacy in complex malignancies like R/M HNSCC. Currently, several promising BsADCs, such as SI-B001 and its derivative BL-B01D1, are undergoing intensive clinical evaluation in this setting.

### 4.1 EGFR×HER3–targeting bispecific ADCs

#### 4.1.1 Izalontamab (SI-B001)

SI-B001 is a novel EGFR×HER3 bispecific antibody comprising an anti-EGFR IgG1 monoclonal antibody fused to two anti-HER3 single-chain variable fragments (scFv) via glycine–serine linkers, designed to block EGFR homodimerization and downstream signaling. A Phase I/Ib study (NCT04603287) evaluating safety and pharmacokinetics enrolled 9 patients (6 with nasopharyngeal carcinoma, 3 with HNSCC). Results showed an ORR of 4% and a DCR of 37%, with a median PFS of 1.9 months. The most common TRAEs included rash (42%), paronychia (25%), and infusion-related reactions (23%), with no treatment-related deaths reported. These findings demonstrate a manageable safety profile and preliminary antitumor activity in advanced HNSCC (**Table 1**) (133).

#### 4.1.2 Izalontamab brengitecan (BL-B01D1)

BL-B01D1 is a bispecific ADC targeting EGFR and HER3, comprising the izalontamab (SI-B001) backbone conjugated to the topoisomerase I inhibitor Ed-04 via a cathepsin-B cleavable tetrapeptide linker, designed to facilitate selective tumor entry and efficient payload release. In a multicenter, randomized, open-label Phase III study involving patients with recurrent or metastatic nasopharyngeal carcinoma refractory to multiple prior therapies, BL-B01D1 demonstrated significantly improved PFS (8.38 months) and ORR (54.6%), with a median DOR of 8.51 months. Common adverse events, including neutropenia, anemia, nausea, vomiting, and fatigue, were predominantly Grade 1–2, with a lower incidence of severe adverse events compared to standard chemotherapy. These findings highlight BL-B01D1 as a potent therapeutic option for refractory NPC, driven by its differentiated EGFR×HER3 dual-targeting mechanism (**Table 1**) (131).

## 5 Summary and future directions

R/M HNSCC exhibits substantial biological and clinical heterogeneity, resulting in limited and often short-lived responses to conventional chemotherapy, targeted therapy, and immune checkpoint inhibitors. Consequently, there remains a significant unmet clinical need for more effective systemic treatment strategies. ADCs, which combine precise tumor antigen targeting with highly potent cytotoxic payloads, have emerged as a promising therapeutic modality capable of addressing these limitations.

To fully appreciate the therapeutic potential of ADCs, it is imperative to define their precise positioning within the evolving treatment landscape of R/M HNSCC. Currently, the frontline standard of care is firmly established by anti-PD-1 immunotherapy (pembrolizumab), either as monotherapy or in combination with platinum-based chemotherapy. However, the majority of patients eventually experience disease progression, leading to a critical “post-immunotherapy bottleneck.” In this heavily pretreated, platinum- and PD-1-refractory setting, traditional salvage options (such as cetuximab, taxanes, or methotrexate) yield notoriously dismal outcomes, with objective response rates plateauing at 10%–15% and a median overall survival of under 6 months. Consequently, the current strategic positioning of ADCs is not to immediately displace frontline immunotherapy, but rather to fill this profound therapeutic void in the post-immunotherapy setting (134). By leveraging precise antigen targeting to deliver potent cytotoxic payloads directly to treatment-resistant tumor cells, ADCs are uniquely positioned as a breakthrough salvage modality for patients who have exhausted standard immuno-oncology and platinum-based regimens.

Accumulating clinical evidence indicates that ADCs targeting TROP-2, EGFR, HER2, HER3, Nectin-4, and B7-H3 can achieve clinically meaningful antitumor activity in heavily pretreated R/M HNSCC, with generally manageable safety profiles. Nonetheless, treatment-related toxicities—particularly hematologic adverse events, interstitial lung disease, and peripheral neuropathy—remain clinically relevant and necessitate careful treatment selection and personalized dose optimization tailored to each patient. As encouraging efficacy has been observed in later-line settings, ongoing efforts are exploring the potential of ADCs in earlier treatment lines and perioperative strategies.

Despite their targeted design, resistance to ADCs represents an emerging challenge, driven by antigen heterogeneity, impaired internalization, and alterations in intracellular drug processing. Rational combination strategies, especially those integrating ADCs with immune checkpoint inhibitors or complementary targeted agents, may help overcome resistance and enhance therapeutic efficacy. Concurrently, continued optimization of ADC architecture—including refined antigen selection, improved linker stability, optimized drug–antibody ratios, and novel payload development—will be critical to maximizing therapeutic benefit while minimizing off-target toxicity.

Another crucial but underexplored clinical dimension is the potential differential efficacy of ADCs between HPV-positive and HPV-negative HNSCC. Given the profound biological dichotomy between these two subtypes, the expression profiles of ADC target antigens can vary significantly. For instance, EGFR overexpression and pathway hyperactivation are hallmark features predominantly associated with HPV-negative HNSCC. Consequently, EGFR-directed ADCs may theoretically exhibit distinct pharmacokinetic accumulation and superior efficacy in this specific cohort. In contrast, emerging preclinical and clinical data suggest that other surface antigens, such as TROP-2, HER2, and HER3, are broadly expressed across both HPV-positive and HPV-negative tumors, potentially broadening the applicability of agents targeting these molecules. Currently, most early-phase ADC clinical trials have enrolled heterogeneous populations with mixed HPV statuses. While objective responses have been observed in both subgroups, robust head-to-head comparisons are hindered by small sample sizes. As the field advances, it is imperative for future phase II/III clinical trials to prospectively stratify R/M HNSCC patients by HPV status. Unraveling whether viral etiology and its associated distinct tumor microenvironment dictate ADC sensitivity will be instrumental in personalizing targeted therapies for HNSCC patients.

Looking forward, a highly promising frontier is the dynamic interplay between ADCs and the characteristically immunosuppressive tumor microenvironment (TME) of HNSCC (135). Beyond their direct targeted cytotoxicity, ADCs can act as potent TME modulators. The targeted delivery of cytotoxic payloads often induces immunogenic cell death, which triggers the release of damage-associated molecular patterns. This process promotes dendritic cell maturation and cytotoxic T-cell activation, effectively reprogramming an immunologically “cold” tumor into an inflamed, “hot” state (136). Furthermore, the antibody backbone of many ADCs retains Fc-mediated effector functions, such as antibody-dependent cellular cytotoxicity. This unique capacity to simultaneously eradicate heterogeneous tumor cells and reverse TME immunosuppression provides a robust mechanistic rationale for the numerous ongoing clinical trials evaluating synergistic combinations of ADCs with immune checkpoint inhibitors.

Finally, advances in biomarker development and patient stratification are essential for the successful clinical integration of ADCs in HNSCC. Standardized detection methods, clearly defined expression thresholds, and deeper insights into tumor-specific antigen biology will facilitate precision medicine approaches. As the ADC landscape continues to evolve, these agents are expected to play an increasingly important role in personalized treatment strategies for patients with R/M HNSCC.

## Glossary

HNC: Head and neck cancer
HNSCC: head and neck squamous cell carcinoma
R/M HNSCC: Recurrent or Metastatic Head and neck squamous cell carcinoma
ADCs: Antibody–drug conjugates
PDX: patient-derived xenograft
CDX: cell line-derived xenograft
HPV: human papillomavirus
TROP-2: Trophoblast cell-surface antigen 2
EGFR: Epidermal Growth Factor Receptor
HER2: Human Epidermal Growth Factor Receptor 2
FDA: U.S. Food and Drug Administration
ICIs: immune checkpoint inhibitors
PD-1: protein 1
T-DM1: ado-trastuzumab emtansine
PDCs: probody–drug conjugates
T-DXd: Trastuzumab deruxtecan
HER3: Human Epidermal Growth Factor Receptor 3
Ror2: Receptor tyrosine kinase–like orphan receptor 2
TF: tissue factor
ITGB6: Integrin Beta-6
SLC3A2: Solute carrier family 3 member 2
ORR: overall response rate
mPFS: median progression-free survival
mDOR: median duration of response
TEAE: treatment-emergent adverse event
SG: Sacituzumab govitecan
MMAE: monomethyl auristatin E
MMAF: monomethyl auristatin F
ABBV-221: Losatuxizumab Vedotin
HER3-DXd: Patritumab deruxtecan
BA3021: Ozuriftamab vedotin
SGN-B6A: Sigvotatug vedotin
CX-2009: Praluzatamab ravtansine
SI-B001: Izalontamab
BL-B01D1: Izalontamab brengitecan
TME: tumor microenvironment
